# Macrophage migration inhibitory factor and angiopoietin-like protein 4 as markers for steroid response in children with idiopathic nephrotic syndrome

**DOI:** 10.1007/s00467-025-06966-0

**Published:** 2025-09-18

**Authors:** Hanaa Al Dash, Heba Mostafa Ahmed, Shireen Ragab Shihatah, Noha Khalifa Abdelghaffar, Mona Gamal Mostafa, Sherin Khamis Hussein

**Affiliations:** 1 Department of Pediatrics, Faculty of Medicine, Fayoum University, Fayoum, Egypt; 2 Department of Pediatrics, Faculty of Medicine, Beni-Suef University, Beni-Suef, Egypt; 3 Department of Clinical Pathology, Faculty of Medicine, Fayoum University, Fayoum, Egypt

**Keywords:** Macrophage migration inhibitory factor, Angiopoietin-like 4, Nephrotic syndrome, Minimal change disease

## Abstract

**Background:**

Idiopathic nephrotic syndrome (INS) is a significant kidney disorder in pediatrics. Early diagnosis of minimal change disease (MCD) is difficult in children with nephrotic syndrome (NS). Angiopoietin-like protein 4 (ANGPTL4), found on the surface of podocytes, has been linked to nephrotic syndrome (NS) and plays a role in triggering proteinuria. Macrophage migration inhibitory factor (MIF) functions as a crucial modulator of the innate immune system and partly counteracts glucocorticoid-induced immune system inhibition. This study aimed to assess the role of ANGPTL4 and MIF as biomarkers in steroid responsiveness of INS.

**Methods:**

This cross-sectional comparative study involved 70 children with NS and 40 healthy children as a control group.

**Results:**

Urinary MIF/creatinine levels were significantly elevated in steroid-resistant nephrotic syndrome (SRNS) relative to in steroid-sensitive nephrotic syndrome (SSNS) and controls (*p* < 0.001). However, ANGPTL4 levels were significantly elevated in the SSNS group relative to the SRNS and control groups (*p* < 0.001). Regarding plasma MIF and urinary MIF/creatinine levels, there were no significant differences between MCD and FSGS, whereas ANGPTL4 levels were significantly elevated in MCD relative to FSGS (*p* < 0.001).

**Conclusions:**

Elevated levels of serum and urinary MIF levels were consistent with SRNS. Furthermore, ANGPTL4 was found to be highly upregulated in SSNS, unlike SRNS, which serves as a potential marker to distinguish between these two diseases.

**Graphical abstract:**

**Figure:**
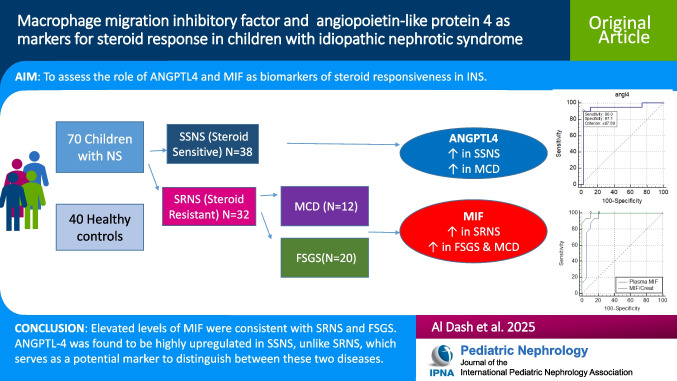
A higher resolution version of the Graphical abstract is available as Supplementary information

**Supplementary Information:**

The online version contains supplementary material available at 10.1007/s00467-025-06966-0.

## Introduction

Idiopathic nephrotic syndrome (INS) is the most frequent pediatric primary glomerular illness, involving 16–17 per 100,000 children aged 2 to 8 years, with the highest prevalence occurring between the ages of 3 and 5 years. INS shows altered permeability of the glomerular filtration barrier, which results in proteinuria [1]. Immune-mediated pathways have been well recognized and researched in glomerular diseases, including membranous nephropathy and glomerulonephritis [2]. Reversible immunological dysregulation is likewise a hallmark of INS, as seen in nongenetic focal segmental glomerulosclerosis (FSGS) and minimal change disease (MCD) [3]. Regrettably, many patients get lengthy but ineffective glucocorticoid (GC) therapy since there are no biomarkers to predict treatment response. This puts these patients who develop steroid-resistant nephrotic syndrome (SRNS) at considerable hazard for both harmful side effects and the advancement of their disease. To forecast clinical steroid responsiveness in glomerular disease, biomarkers have been developed through cytokine profiling and through directed approaches such as gene arrays, enzyme-linked immunosorbent assay testing, or other approaches like proteomics, metabolomics, and transcriptomics [4].

Despite these attempts, we still do not understand the molecular mechanisms that regulate glucocorticoid resistance in NS, and there are no serum, urine, or salivary biomarkers that can predict it [5]. MIF is an essential innate immunity regulator, termed glycosylation-inhibiting factor (GIF) [6]. Macrophage migration inhibitory factor (MIF) is categorized as an inflammatory mediator, as bacterial antigens cause leukocytes to release MIF into the circulation. MIF attaches to CD74 on other immune cells, causing an acute immunological response. Furthermore, GC encourages leukocytes to secrete MIF, which partially counteracts glucocorticoids’ immunosuppressive effects [7]. The angiopoietin-like protein family (ANGPTL1–8) includes angiopoietin-like protein 4 (ANGPTL4). ANGPTL4 appears to perform pro- or anti-inflammatory functions in various tissues and illnesses, making it difficult to create ANGPTL4-targeting therapeutics. It is triggered by interleukin-1β (IL-1β) and plays a role in various inflammatory disorders. Exposure to prostaglandins-b, interferon-γ, tumor necrosis factor-α, and interleukins has led to increased levels of ANGPTL4 in 3T3L1 adipocytes [6, 8]. Recent studies have indicated that podocyte-secreted ANGPTL4 causes proteinuria, while circulating ANGPTL4 contributes to hypertriglyceridemia [9, 10]. This research aims to examine the role of serum ANGPTL4, MIF, and urinary MIF as predictors for steroid response and underlying pathology in INS in children.

## Patients and methods

**Fig. 1:**
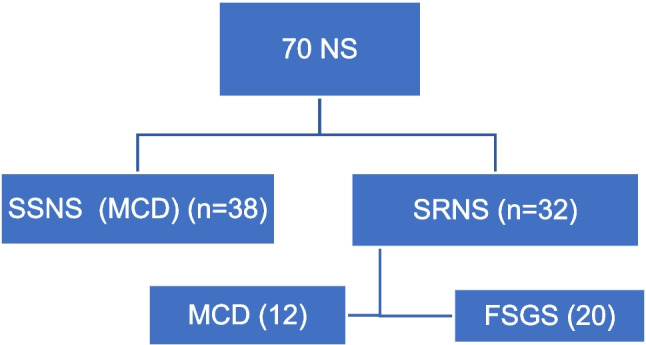
Flow chart of the study population

This was a cross-sectional case-control study undertaken from October 2022 to April 2024 involving 110 children allocated into three groups: Group 1, Steroid-Sensitive Nephrotic Syndrome (SSNS) with 38 patients; Group 2, Steroid-Resistant Nephrotic Syndrome (SRNS) with 32 patients; and Group 3, Healthy Controls with 40 children recruited from the general outpatient clinic and healthy siblings of cases (Fig. 1). To study the effect of steroids and calcineurin inhibitors (CNI) on marker levels, patients in either group were subdivided into a remission group while receiving treatment and a relapse group (relapse after withdrawal of immunosuppressive therapy). Furthermore, patients were classified as MCD and FSGS according to the results of kidney biopsy. Children who had SSNS aged between 1 and 6 years without hypertension, hematuria, or low estimated glomerular filtration rate (eGFR) were considered MCD. The inclusion criteria were children diagnosed with NS ranging from 1 to 18 years old. The criteria for exclusion were based on the onset of NS before reaching 1 year of age, the presence of acute infections during the 3 months prior to enrollment in the study, an eGFR of less than 60 ml/min/1.73 m^2^, and the existence of secondary NS (e.g., SLE, Alport syndrome). All children had a comprehensive medical history taken, which included their age, age at disease onset, duration of the disease, response to steroid therapy, other immunosuppressive treatments, results from kidney biopsy, antihypertensive medications, and a family history of conditions affecting their siblings. Clinical examination included weight in kilograms (kg), height in centimeters (cm), and body mass index (BMI), which was computed employing the standard formula (body mass in kilograms divided by the square of body height in meters). Diastolic and systolic blood pressures, along with the presence of edema and a comprehensive systemic examination, were noted. Routine laboratory investigations included complete blood count, urinary protein/creatinine ratio (Ptn/Creat ratio), kidney function tests (urea and creatinine), serum albumin, serum ANGPTL4, serum MIF, and urinary MIF. The caregivers of the participating children provided informed consent, and the research was authorized by the local ethics commission.

### Study definitions

Relapse: 24 h urinary protein more than 40 mg/m^2^/h or urinary protein more than 3 + by dipstick in spot sample for at least three consecutive days. Remission: 24 h urinary protein less than 4 mg/m^2^/h, nil or trace by dipstick for at least three consecutive days. Frequent relapses: Two or more relapses within 6 months of steroid-induced remission; 4 or more relapses in any 12 months. Steroid dependence: at least 2 consecutive relapses during cortisone treatment with or within 2 weeks of its withdrawal. Steroid resistance: Failure to achieve remission after 4–8 weeks of daily oral prednisolone therapy at a dose of 2 mg/kg/day [11].

## Sample collection

The blood and urine of participants were collected prospectively in plain tubes, then centrifuged and kept at − 80 °C until analysis. Once finished, serum concentrations of ANGPTL4 and MIF, along with the urinary excretion of MIF, were determined employing enzyme-linked immunosorbent assay (ELISA) kits from Bioassay Technology Lab (Cat. Nos. E3119Hu and E0141Hu, respectively, China).

## Statistical methodology

Information gathered from patient history, fundamental clinical assessments, lab investigations, and outcome evaluations was recorded, organized, and analyzed using Microsoft Excel. The data were afterward input into the Statistical Package for the Social Sciences (SPSS version 20.0) program for analysis. The categorical data were expressed as number and percentage, and differences among groups were analyzed employing the chi-squared test. ANOVA and *T*-tests were applied to compare continuous variables (mean ± SD) between groups, while Pearson’s correlation test was utilized to establish correlations. *P*-values < 0.05 were interpreted as statistically significant.

## Results

The mean age of the patient group was 9.29 ± 3.22 years (ranging from 2 to 14 years), with 68% (48) being male versus 70% (28) in the control group, and the mean age at onset was 5 ± 2.5 years (from 1.5 to 10 years). The mean disease period was 3.27 ± 2.58 years (ranging from 0.6 to 12 years). Thirty-two patients were SRNS, and all of them underwent kidney biopsy. MCD (based on clinical criteria, response to steroids, and kidney biopsy in SRNS cases) was reported in 50 patients, while FSGS was reported in 20 patients. 38 (76%) of the MCD patients did not receive immunosuppressants rather than steroids, while 12 patients received cyclosporine. In the FSGS group, 18 (90%) received cyclosporine and 2 (10%) received tacrolimus. A comparison between cases and controls is presented in Table 1. The two marker levels were significantly elevated in cases relative to controls. Comparisons between SRNS and SSNS are shown in Table 2. Urinary MIF/creatinine levels were significantly elevated in the SRNS group relative to the SSNS group and controls (*p* < 0.001), while no significant distinctions were documented among the SSNS group and controls. In contrast, ANGPTL4 levels were significantly elevated in the SSNS group relative to the SRNS group and controls (*p* < 0.001) without significant differences among the SRNS group and controls (*p* > 0.05). When stratifying both groups according to disease activity (SSNS in remission and SSNS in relapse, SRNS in remission and SRNS in relapse) we found that the levels of uMIF/creat and plasma MIF remained significantly higher in the SSNS groups compared to the SRNS groups, while the ANGPTL4 levels were significantly higher in the SRNS subgroups (Table 3). Laboratory data from the MCD and FSGS groups in the SRNS group are shown in Table 4. Regarding serum and urinary MIF/creatinine levels, there were no notable differences among MCD and FSGS. On the other hand, ANGPTL4 levels were significantly increased in MCD relative to FSGS (*p* < 0.001).

**Table 1:** Comparison between the cases and controls regarding clinical and laboratory data

Group	Cases (*n* = 70)	Controls (*n* = 40)	*p*
Age (years)	9.29 ± 3.22	7.65 ± 2.92	0.066
Sex: male/female	48/22	28/12	0.89
Weight (kg)	28.71 ± 7.97	25.90 ± 7.64	0.207
Height (cm)	130.91 ± 18.05	128.10 ± 18.25	0.582
BMI (kg/m^2^)	16.42 ± 1.30	15.47 ± 0.91	0.005
Systolic BP (mmHg)	100.86 ± 8.79	94.50 ± 8.41	0.011
Diastolic BP (mmHg)	67.71 ± 4.90	65.00 ± 3.97	0.040
Urea (mg/dl)	29.91 ± 7.95	23.50 ± 8.20	0.006
Creatinine (mg/dl)	0.43 ± 0.16	0.24 ± 0.09	< 0.001
eGFR (ml/min/1.73 m^2^)	112.61 ± 22.34	123.23 ± 34.45	0.042
Albumin (g/dl)	4.16 ± 0.45	4.34 ± 0.28	0.110
uPtn/Creat ratio (mg/g creatinine)	227.54 ± 175.65	104.80 ± 34.91	0.003
Hb (g/dl)	11.09 ± 1.16	10.71 ± 1.29	0.269
WBCs (× 10^3^cell/cmm)	12.17 ± 2.74	10.49 ± 1.59	0.015
Platelets (× 10^3^cell/cmm)	353.37 ± 113.51	340.70 ± 96.30	0.676
Urinary MIF (pg/ml)	1002.38 ± 1039.24	472.92 ± 210.67	0.029
uMIF/creat (pg/g)	802.86 ± 299.08	398.00 ± 61.18	< 0.001
Plasma MIF (pg/ml)	749.14 ± 416.83	286.00 ± 20.10	0.002
ANGPTL4 (pg/ml)	119.65 ± 98.67	93.80 ± 43.30	< 0.001

*p*-value > 0.05 (non-significant)

*eGFR* estimated glomerular filtration rate, *uPtn/create* urinary protein/creatinine ratio, *Hb* hemoglobin, *WBCs* white blood cells, *MIF* macrophage migration inhibitory factor,* ANGPTL4* angiopoietin-like protein 4

**Table 2:** Comparison between the SSNS, SRNS, and control groups regarding age, sex, and laboratory data

	SSNS (38)	SRNS (32)	Controls (40)	***p***	
Age (years)	8.55 ± 3.22	10.15 ± 3.07	7.65 ± 2.92	0.14	
Sex: male/female	28/10	20/12	28/12	0.67	
Urea (mg/dl)	27.25 ± 6.46	33.77 ± 8.06	23.50 ± 8.20	p1	0.12
				p2	0.09
				p3	0.02
Creatinine (mg/dl)	0.35 ± 0.14	0.50 ± 0.18	0.24 ± 0.09	p1	< 00.1
				p2	0.01
				p3	< 0.001
eGFR (ml/min/1.73 m^2^)	122.56 ± 23.98	109.49 ± 24.82	123.23 ± 34.45	p1	0.01
				p2	0.81
				p3	0.01
Albumin (g/dl)	4.07 ± 0.37	4.28 ± 0.54	4.34 ± 0.28	0.203	
Ptn/Creat ratio (mg/g creatinine)	161.75 ± 44.96	305.46 ± 235.79	104.80 ± 34.91	p1	< 0.001
				p2	0.08
				p3	< 0.001
Plasma MIF (pg/ml)	467.50 ± 317.83	1090.68 ± 715.41	286.00 ± 20.10	p1	0.001
				p2	0.47
				p3	< 0.001
Urinary MIF (pg/ml)	664.87 ± 394.22	1408.74 ± 601.37	472.92 ± 210.67	p1	0.02
				p2	0.7
				p3	0.03
uMIF/creat (pg/g)	570.17 ± 120.42	1079.23 ± 175.95	398.00 ± 61.18	p1	< 0.001
				p2	< 0.001
				p3	< 0.001
Plasma ANGPTL4 (pg/ml)	186.67 ± 82.2	98.67 ± 41..53	93.80 ± 43.30	p1	< 0.001
				p2	< 0.001
				p3	0.67

p1, comparison between SSNS and SRNS

p2, comparison between SSNS and controls

p3, comparison between SRNS and controls

**Table 3:** Comparison between the SSNS and SRNS groups while in remission and in relapse

	Relapse group			Remission group		
	SSNS (*n* = 15)	SRNS (*n* = 10)	*p*	SSNS in remission (*n* = 23)	SRNS in remission (*n* = 22)	*p*
uMIF/creatinine (pg/g)	570.17 ± 120.42	1079.23 ± 175.95	< 0.001	662. 45 ± 98.41	1115.18 ± 103.61	< 0.001
Plasma MIF (pg/ml)	398.59 ± 210.76	934.56 ± 128.87	< 0.001	519.39 ± 193.78	1121.03 ± 430.19	< 0.001
Plasma ANGPTL4 (pg/ml)	165.38 ± 53.09	89.29 ± 36.62	< 0.001	173.52 ± 98.11	103.89 ± 44.91	0.004

*uMIF/creatinine* urinary macrophage migration inhibitory factor/creatinine ratio, *ANGPTL4* angiopoietin-like protein 4

**Table 4:** Comparison between FSGS and MCD in the SRNS group regarding laboratory data

	FSGS (20)	MCD (12)	*p*
Albumin (g/dl)	4.27 ± 0.60	4.25 ± 0.36	0.943
Ptn/Creat ratio (mg/g creatinine)	350.90 ± 287.18	231.00 ± 85.27	0.342
Urea (mg/dl)	32.40 ± 8.11	34.17 ± 8.50	0.685
Creatinine (mg/dl)	0.50 ± 0.19	0.48 ± 0.15	0.789
eGFR (ml/min/1.73 m^2^)	107.68 ± 21.67	111.49 ± 18.88	0.58
Urinary MIF (pg/ml)	1512.95 ± 1438.28	1234.31 ± 933.54	0.680
MIF/creat	1030.00 ± 177.14	1161.67 ± 189.36	0.182
Plasma MIF (pg/m)l	1265.00 ± 864.89	800.00 ± 168.76	0.220
ANGPTL4 (pg/ml)	73.46 ± 32.22	184.55 ± 79.09	< 0.001

*eGFR* estimated glomerular filtration rate, *uPtn/create* urinary protein/creatinine ratio, *Hb* hemoglobin, *WBCs* white blood cells, *MIF* macrophage migration inhibitory factor, *ANGPTL4* angiopoietin-like protein 4

**Fig. 2:**
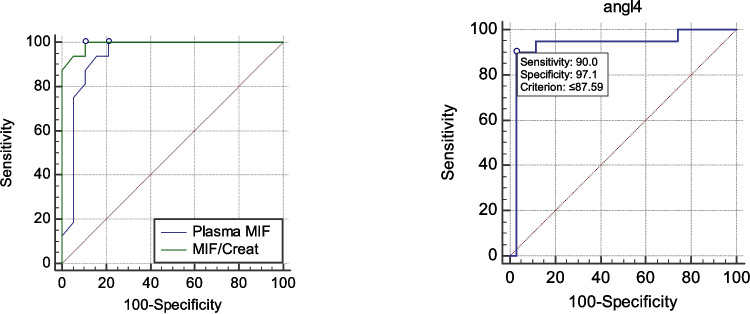
ROC curves for MIF and ANGPTL4 to discriminate between SSNS and SRNS

The ROC analysis revealed that ANGPTL4 can differentiate between SSNS and SRNS at a cutoff of < 87.5 pg/ml with a sensitivity of 90%, a specificity of 97%, and an AUC of 0.93, whereas a serum MIF cutoff value of > 490 pg/ml had a sensitivity of 100%, a specificity of 87%, and an AUC of 0.94%. Similarly, uMIF/creatinine can discriminate between the two groups at a cutoff value > 660 pg/mg with a sensitivity of 100%, a specificity of 90%, and an AUC of 0.99% (Fig. 2).

## Discussion

Idiopathic nephrotic syndrome is the predominant kind of NS, with the remaining etiologies being secondary to other conditions such as congenital, infections, or glomerular diseases. There are numerous theories in the literature on the pathogenesis of INS, attributed to various mechanisms such as glomerular abnormalities, cytokines, or immunological problems [12]. In this case-control study, we wanted to study the role of serum MIF and urinary MIF as predictive markers for INS steroid responsiveness, as well as their utility in understanding the underlying pathology, steroid resistance mechanism, and potential therapeutic targets. In terms of serum MIF, we found a significantly elevated level in the SRNS group relative to the SSNS group (*p* = 0.001). Cuzzoni et al. reported that serum MIF levels were markedly elevated in the setting of steroid-resistant INS, with a mean concentration of 759.7 pg/mL, compared to steroid-sensitive INS and steroid-dependent INS, with a concentration of 414.1 pg/mL (*p* = 0.022). Concurring with our findings, they proposed that serum MIF could be of use as a biomarker to predict responsiveness to steroid therapy in children with INS [5]. Ramayani et al. also put forth data supporting our proposition, as they found that children who had SRNS had a genetic mutation in the gene *MIF-173G/C*, with the C allele being frequently met in the SRNS group relative to the SSNS and healthy controls (*p* = 0.025), and they also noted that serum MIF levels were significantly increased above 20 ng/mL in the SR group (*p* = 0.04) as well as in the group with the C allele (*p* = 0.04), which suggests that the variability in expression of MIF may have a genetic basis and may determine response to steroid in INS [13]. In contrast to our findings, Zwiech et al. concluded that levels of serum MIF were, indeed, significantly increased in subjects with glomerulonephritis (whether responsive or not to immunosuppressive treatment) in contrast to healthy controls (*p* = 0.036); however, the levels did not differ significantly between steroid-responsive and steroid-resistant cases, whether at baseline or after initiation of immunosuppressive treatment [14]. Our findings can be attributed to the fact that MIF is a proinflammatory cytokine that induces the chemotaxis of macrophages and T cells, evoking an inflammatory response, adding to the fact that it antagonizes the immunosuppressive effect of glucocorticoids—despite being released by glucocorticoids during a stressful kidney event, such as acute kidney injury—thus, it causes an inflammatory process in the kidney leading to activation of macrophages, which subsequently causes the release of more MIF, and a vicious cycle is established [15–17]. Therefore, patients who are impacted by steroid-resistant kidney disease may have favorable outcomes if a blocking agent targeting MIF is used instead of glucocorticoids [18]. Reflecting on urinary MIF, we demonstrated a significant rise in the SRNS group as opposed to the SSNS group (*p* = 0.035). This could be explained by the fact that the diseased kidney tissues express MIF at dramatic levels, which raises the serum levels of MIF, and consequently, due to the underlying glomerulonephritis, a urinary output impairment ensues, leading to higher excretion of MIF in the urine. Zwiech et al. supported this finding as they observed that urinary MIF was substantially higher in primary glomerulonephritis versus healthy controls, irrespective of the type of primary GN (*p* = 0.006, *p* = 0.009). They also noted that following treatment, the levels of urinary MIF were significantly greater in the steroid non-responders than in those who responded to treatment (*p* < 0.05) [9]. Endorsing our findings, Lan et al. discerned that, according to immunohistochemical analysis, MIF was a normal constituent of kidney tissues but highly upregulated in the case of glomerulonephritis, and they inferred that kidney expression of MIF was much more prominent in areas of tissue damage when compared with the histochemical appearance of other areas without damage, and they owed that to macrophage (*p* < 0.0001) and T cell (*p* < 0.05) release of MIF at areas of focal damage, not to the normally expressed MIF by glomerular or tubular cells of the kidney. Overall, they substantiated our data by confirming that elevated urinary MIF was, in fact, mainly due to active inflammation and that urinary MIF could serve as a potential marker of kidney inflammation and a potential therapeutic target in patients who fail to respond to steroids [19]. Others, such as Kong et al., reported ambivalent results that contrast with our results; Kong et al.’s results hint that the elevated urinary MIF levels may be attributed to MIF expression by intrinsic cells of the kidney (podocytes, tubular cells, endothelial cells, and mesangial cells), along with resident macrophages, T lymphocytes, and fibroblasts [20]. Additionally, the SRNS group had a significantly greater MIF/creatinine ratio (*p* < 0.001).

The ROC analysis of our data regarding serum MIF showed that at a cutoff score of > 490 pg/ml, serum MIF could discriminate between SSNS and SRNS with a sensitivity of 100% and a specificity of 87% (AUC = 0.936). Our analysis and deductions are supported by Cuzzoni et al., who observed that patients with SRNS had higher levels of serum MIF and concurred that at a cutoff of > 501 pg/ml, MIF could significantly identify resistance to steroid therapy with a sensitivity of 85.7% and a specificity of 71.4% [5]. In our study, we intended to evaluate the role of ANGPTL-4, a protein expressed in various tissues of the body, including endothelial cells and podocytes, in the context of INS. Intriguingly, we noted that serum ANGPTL-4 levels were significantly higher in the SSNS group (186.67 pg/ml) as opposed to the SRNS group (73.46 pg/ml, *p* = 0.000), but insignificantly higher than in the control group. Li et al. experimented on nephrotic mice, comparing those without the *ANGPTL-4* gene (using the CRISPR/Cas9 technique to eliminate the *ANGPTL-4* gene) with mice that still had the gene, in terms of proteinuria and hyperlipidemia. They found that lipid disorders and podocyte effacement were relieved in mice with the knocked-out *ANGPTL-4* gene, with significantly lower proteinuria and substantially reduced total cholesterol and triglyceride levels as opposed to nephrotic mice with the gene, underlining the role of ANGPTL-4 in both the pathophysiology of NS and the associated hyperlipidemia. Furthermore, they knocked out the gene in the ANGPTL4 +/+ mice, which then displayed notable relief of severe proteinuria and amelioration of disordered lipid metabolism [21]. Following our results, Clement et al. addressed the function of ANGPTL-4 in nephrotic syndrome by injecting ANGPTL4 +/+ rats with a nephrotoxic agent, and they noted a significant elevation in the levels of ANGPTL4 mRNA in these rats. However, when ANGPTL4-/- rats were injected with the same nephrotoxic agent, the levels of proteinuria and podocyte effacement were substantially lower. Moreover, they studied the possible contribution of sialylation in the variable response of tissues to ANGPTL-4 and showed that nephrotic rats that were treated with ManNAc (a sialylating agent) showed much lower levels of proteinuria compared to baseline. In addition to that, they interrogated the sensitivity of ANGPTL-4 to glucocorticoids and concluded that this characteristic significantly favors MCD and other SSNS, as glucocorticoids seem to significantly increase the expression of the sialylated form of ANGPTL-4, leading to amelioration of proteinuria [9]. This could be explained by the fact that ANGPTL-4 acts on various tissues of the body in multiple ways, some of which induce podocyte injury and proteinuria and inactivation of lipoprotein-lipase (LPS) [22]. A hyposialylated form of ANGPTL-4 is expressed exclusively by podocytes of the glomeruli, inducing proteinuria. This hyposialylated form then moves into the bloodstream, which is then expressed, after being sialylated, by other tissues like adipose tissue, cardiomyocytes, and skeletal muscles, and with treatment with glucocorticoids in steroid-sensitive nephrotic syndrome, this form of ANGPTL-4 is expressed even more by peripheral tissues [23]. The sialylated form of ANGPTL-4 then binds to glomerular endothelial cells and reduces proteinuria but also inhibits LPS, which causes hyperlipidemia [24]. This explains the partial response to steroids in some types of nephrotic syndrome, as well as the steroid-dependent types. This approach in treatment leads to activation of the systemic and local feedback loop of ANGPTL-4 expression, which then worsens hyperlipidemia of nephrotic syndrome and leads to unsatisfactory responsiveness of proteinuria [25]. We further evaluated the treatment groups again after subgrouping based on the type of nephrotic syndrome into the FSGS group and MCD group. We determined that there were no statistically significant differences between both groups regarding all parameters except ANGPTL-4 levels, which were higher in the MCD group (184.55 pg/ml) in contrast to 73.46 pg/ml in the FSGS group. We deemed this to be of statistical significance (*p* < 0.001). This aligns with the results of Clement et al., who also noted that podocytes in both human and animal model MCD expressed ANGPTL-4 much more than podocytes in other kidney diseases such as membranous nephropathy, with unchanged ANGPTL-4 levels in focal segmental glomerulosclerosis (FSGS). Furthermore, they noted that ANGPTL-4 oligomers could be found in the urine of patients with MCD, as well as upregulated in their serum, unlike those with FSGS. They also stated that ANGPTL-4, being a steroid-sensitive molecule, has a significant contribution to the development of MCD, as its expression by podocytes results in a filtration defect, which in turn leads to nephrotic-range proteinuria. To confirm these results, they experimented on NHPS2-ANGPTL4 transgenic rats and extrapolated that the ANGPTL-4, via complex interplay with the podocyte-GBM interface, results in foot process effacement, and that mutation of the *NHPS2* gene, which encodes the podocyte protein podocin, results in a steroid-resistant variant of nephrotic syndrome, namely FSGS [9]. Li et al. also reported on the role of ANGPTL-4 in the development of INS and indicated that glomerular expression of ANGPTL-4 was only notable in MCD, unlike MSPGN and FSGS [26]. This can be explained by the fact that ANGPTL-4 is a glucocorticoid-sensitive protein that is heavily upregulated in the kidney as well as peripheral tissues in the context of steroid-sensitive kidney disease, and due to being enacted through interaction with podocyte integral proteins such as podocin, which are regulated by genes such as *NHPS2*, mutations of these genes might be responsible for steroid-resistant nephrotic syndrome like FSGS, which might explain the significantly lower ANGPTL-4 expression in FSGS compared with its upregulation in MCD [9, 27–29].

### Study limitations

This study has several limitations, including the relatively small sample size and the inability to study more biomarkers due to the lack of funding. Also, we were unable to measure the markers at the onset of the first attack in all of the patients.

## Conclusion

In conclusion, our study addressed the role of MIF and ANGPTL-4 in the pathogenesis and management of nephrotic syndrome, as well as their position as determinants of steroid responsiveness of glomerular diseases. We presented our data, backed by the available literature, which inferred that elevated levels of serum and urinary MIF were consistent with SRNS, which endorses the hypothesis that MIF could potentially act as a biomarker for steroid-resistant INS. Furthermore, we touched upon ANGPTL-4, which, in contrast, was found to be highly upregulated in SSNS, unlike SRNS, which serves as a potential marker to distinguish between these two disease states. We elaborated on the relation between glucocorticoids and ANGPTL-4, highlighting the relevance of it being a steroid-sensitive protein in the context of MCD versus FSGS. Our data put forth the proposition that not only could MIF and ANGPTL-4 have a significant function in the understanding of the etiopathogenesis of INS and predict the responsiveness of disease early on, but also they may offer valuable alternatives to steroids and immunosuppressive agents in the treatment of steroid-resistant and steroid-dependent disease using targeted therapeutic approaches.

## Supplementary Information

Below is the link to the electronic supplementary material.

Graphical abstract (PPTX 96 KB)

## Data Availability

The data employed in the current research can be requested from the corresponding author with justification.
