# Supplementary material for: Macrophage migration inhibitory factor and angiopoietin-like protein 4 as markers for steroid response in children with idiopathic nephrotic syndrome
Source: Pediatr Nephrol. 2025 Sep 18;41(2):391–8. doi: 10.1007/s00467-025-06966-0 (PMC12727841; doi:10.1007/s00467-025-06966-0)
Supplement: Supplementary file 1 — Graphical abstract (PPTX 96 KB) [file 467_2025_6966_MOESM1_ESM.pptx]

## Slide 1
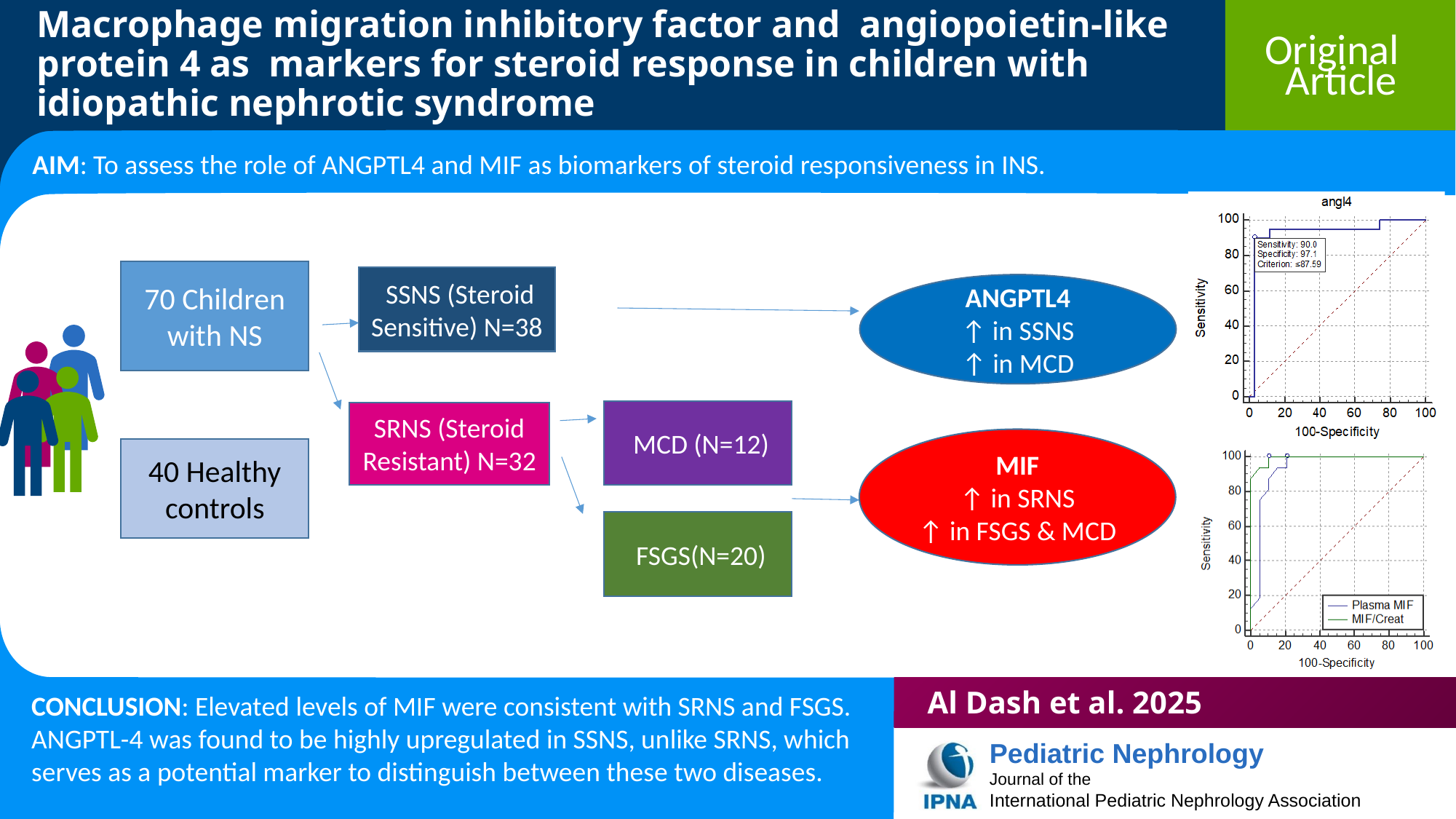

Macrophage migration inhibitory factor and angiopoietin-like protein 4 as markers for steroid response in children with idiopathic nephrotic syndrome
AIM: To assess the role of ANGPTL4 and MIF as biomarkers of steroid responsiveness in INS.
70 Children with NS
 SSNS (Steroid Sensitive) N=38
ANGPTL4
↑ in SSNS
↑ in MCD
 MCD (N=12)
SRNS (Steroid Resistant) N=32
MIF
↑ in SRNS
↑ in FSGS & MCD
40 Healthy controls
 FSGS(N=20)
Al Dash et al. 2025
CONCLUSION: Elevated levels of MIF were consistent with SRNS and FSGS. ANGPTL-4 was found to be highly upregulated in SSNS, unlike SRNS, which serves as a potential marker to distinguish between these two diseases.
